# Application of *Ascophyllum nodosum*-Based Soluble Extract on Micropropagation and Regeneration of *Nicotiana benthamiana* and *Prunus domestica*

**DOI:** 10.3390/plants10071354

**Published:** 2021-07-02

**Authors:** Mohamed Faize, Lydia Faize, Lorenzo Burgos, Alan T. Critchley, Nuria Alburquerque

**Affiliations:** 1 Laboratory of Plant Biotechnology, Ecology and Ecosystem Valorization, Faculty of Sciences, University Chouaib Doukkali, El Jadida 24000, Morocco; faizemohamed@yahoo.fr; 2 Group of Fruit Tree Biotechnology, Department of Plant Breeding, CEBAS-CSIC, P.O. Box 164, 30100 Murcia, Spain; lbremaud@cebas.csic.es (L.F.); burgos@cebas.csic.es (L.B.); 3 Verschuren Centre for Sustainability in Energy and the Environment, Sydney, NS B1P 6L2, Canada; alan.critchley2016@gmail.com

**Keywords:** Morphogenesis, shoot regeneration, rhizogenesis, in vitro culture, biostimulants, seaweed extract

## Abstract

In the present study, the effect of a commercial extract of the seaweed *Ascophyllum nodosum* on in vitro micropropagation, shoot regeneration, and rhizoghenesis were studied in *Nicotiana benthamiana* and *Prunus domestica*. Results showed that the MS medium supplemented with various concentrations of the *Ascophyllum* extract (5, 10, 50, and 100 mg L^−1^) significantly enhanced the number of regenerated buds from *N. benthamiana* leaf discs to the conventional MS regenerating medium. Increases ranged from 3.5 to 6.5 times higher than the control. The effect of the *Ascophyllum* extract on *N. benthamiana* micropropagation was assessed through the measurement of some plant growth parameters. Results showed that the extract alone could not replace the micropropagation medium since shoot length, shoot diameter, root length, and leaf area were significantly reduced. However, its combination with a half-strength MS medium enhanced these parameters. Its effect was also evaluated on regeneration from plum hypocotyl slices. When added to the shoot regeneration medium without any plant growth regulators, the *Ascophyllum* extract alone could induce shoot regeneration. However, the percentage of bud regeneration and number of regenerated buds were lower than with the conventional shoot regeneration medium containing complete growth regulators. In contrast, the *Ascophyllum* extract drastically promoted rhizogenesis from plum hypocotyl slices. These results pave the way for the possible use of *A. nodosum* extracts in in vitro mass propagation of higher plants.

## 1. Introduction

Biostimulants are any substance, microorganism, or material, apart from nutrients and pesticides, that can positively affect plant growth [1]. Their use has increased dramatically over the past decade [2]. Among them are those derived from intertidal seaweed are widely used in the agriculture industry because of the presence of several groups of plant promoting substances and diverse substances known to positively affect stress signaling and provide beneficial effects on the growth parameters and biomass of different crops [3,4]. In this regard, extracts from the brown seaweed *Ascophyllum nodosum* have been reported to improve plant growth and to alleviate abiotic and biotic disorders by improving plant defenses [5]. Several companies worldwide are involved in manufacturing extracts from *A. nodosum* for agricultural and horticultural applications. Commercial extracts from *A. nodosum* have been reported to enhance the growth of vegetables such as lettuce and spinach [6,7] and to improve the fruit quality of watermelons, apples, and grapes [8,9,10]. They enhanced the macronutrient and micronutrient contents in tomato fruits, olive plants, and grapevines [11,12,13].

Several studies have demonstrated that the application of *A. nodosum* extracts alleviated abiotic stresses such as drought in *Arabidopsis thaliana*, tomato, bean, soybean, spinach, and sweet orange by enhancing osmolyte accumulation, stomatal regulation, and improvement of antioxidant defenses [14,15,16,17,18]. The application of *A. nodosum* extracts also alleviated the negative effect of salinity stress in *Arabidopsis* and tomato [11,19]. In avocado, salt stress tolerance was paralleled with elevated Ca^2+^ and K^+^ [20]. Bioactive compounds present in *A. nodosum* are also considered as potent elicitors of plant defense responses against various pathogens [21,22,23]. When alternated with metalaxyl fungicide, the *A. nodosum* extracts improved plant disease resistance against *Phytopthora melonis* in cucumber [24] and against *Phytophthora capsica* in tomato [25]. Disease resistance was correlated with the enhancement of the activity of several enzymes involved in plant defense responses including peroxidase (PO), polyphenol oxidase (PPO), lipoxygenase, phenylalanine ammonia lyase (PAL), and β-1,3-glucanase (Glu). Jayaraj et al. [26] showed that a foliar spray of ANE to carrot plants reduced the progression of disease caused by *Alternaria radicina* and *Botrytis cinerea* by priming the activity of PO, PPO, PAL, chitinase, and Glu as well as increasing the transcript accumulation of NPR1 and various pathogenesis related proteins (PR) in carrot. Extracts from *Cystoseira myriophlloides*, *Fucus spiralis*, and *Laminaria digitata* were effective in reducing *Verticillium* wilt and crown gall disease severity in tomato and wild fire disease in *Nicotiana benthamiana* [27,28].

Because of their richness in macronutrients, micronutrients [3] and growth regulators [29,30], intertidal seaweed extracts have been used as additives for improving the tissue culture of higher plants. For instance, when compared to conventional growth regulators, extracts from *Sargassum wightii* promoted in vitro shoot elongation and rooting in tomato [31]. When supplemented with low doses of plant growth regulators, various extracts deriving from *Padina gymnospora* and *Padina boergesenii* enhanced in vitro shoot proliferation, and rooting of shoots from hypocotyls and leaf disc explants in eggplant [32]. Similarly, when liquid extracts from *Cystoseira myriophylloides* or *Fucus spiralis* were used separately, they improved regeneration from tobacco leaf discs and in vitro micropropagation of grapevine, plum, and apricot shoots [33].

The commercial extract from the brown seaweed *Ascophyllum nodosum* (AE) described by Hurtado et al. [34] has been widely traded for agricultural farming purposes. It has also been reported for its use in in vitro micropropagation of algae. When combined with the plant growth regulators at lower concentrations, it boosted the micropropagation of *Kappaphycus alvarezii* [34] and of the agarophyte *Gracilaria blodgetti* [35]. However, apart from *A. thaliana*, which has been used as a rapid bioassay, to the best of our knowledge *Ascophyllum nodosum* extracts have never been tested for the micropropagation and regeneration of other higher plants. In this context, the present investigation was carried out to determine the effect of AE on the in vitro regeneration, rooting, and micropropagation of the herbaceous plant *Nicotiana benthamiana* and the woody plant *Prunus domestica*.

## 2. Results

### 2.1. Analysis of Plant Growth Regulators Composition of Ascophyllum Nudosum Extract

Results showed that AE contained some plant growth regulators (Table 1). AE has relatively low concentrations of cytokinins; only 2-isopentenyl adenine (2-iP) was found at 0.47 ng mL^−1^. Concentration of giberellin GA4 was 0.11 ng mL^−1^ and indole-3-acetic acid (IAA) was not detected in AE, abscisic acid (ABA) was present in AE at 0.52 ng mL^−1^, and relatively high amounts of salicylic acid (SA) (10.20 ng mL^−1^) were detected in AE.

### 2.2. Effect of Ascophyllum Nodosum Extract on In Vitro Micropropagation of Nicotiana benthamiana

Different concentrations of AE ranging from 20 to 100 mg L^−1^ alone or mixed with a half-strength MS medium (MS/2) were used for micropropagation of *N. benthamiana* plantlets and compared to the MS/2 medium (Table 2). Shoot length was significantly reduced in plantlets cultivated in the presence of AE alone, regardless of the concentration. However, a combination of AE at concentrations ranging from 20 to 80 mg L^−1^ with MS/2 resulted in higher shoots when compared to those grown on the MS/2 medium. Shoot diameter was significantly reduced in plantlets grown in the presence of AE alone and in MS/2 mixed with 80 or 100 mg L^−1^, the highest AE concentrations tested. The rest of the combinations did not significantly enhance shoot diameter. Similarly leaf area was reduced when plantlets were grown in AE alone and combined 100 mg L^−1^. While AE alone at 40, 60, 80, and 100 mg L^−1^ reduced root length, most combinations of AE with MS/2 did not significantly affect this parameter, and MS/2 + 100 mg L^−1^ enhanced this parameter.

As a whole, these results show that AE alone is not sufficient to promote the growth of *N. benthamiana* plantlets in vitro, while its combination with MS/2 slightly improved some growth parameters such as shoot or root length.

### 2.3. Effect of Ascophyllum nodosum Extract on Regeneration of Nicotiana benthamiana

**Figure 1:**
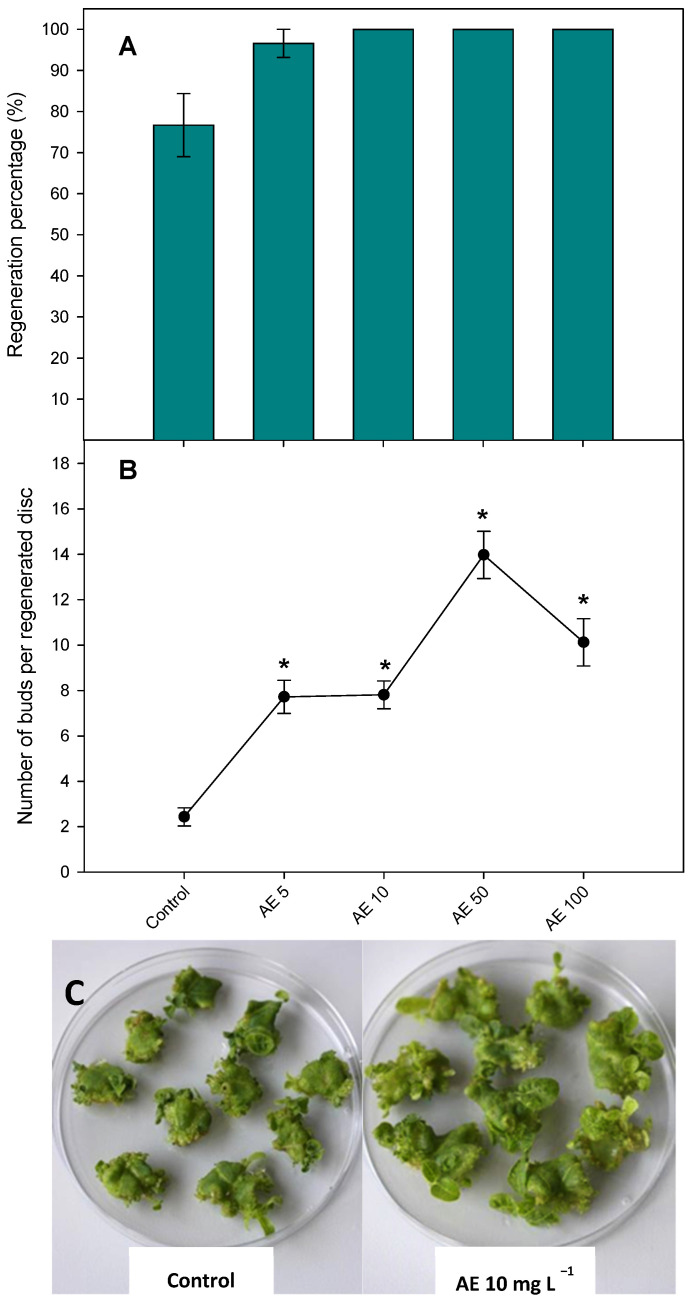
Effects of *Ascophyllum nodosum* extract (AE) at 5, 10, 50, and 100 mg L ^−1^ on the percentage of regeneration (**A**) and the number of buds per regenerated explant from *Nicotiana benthamiana* leaf discs (**B**). (**C**) Photo showing shoot regeneration. Data are percentages ± standard errors in (**A**) and means ± standard errors in (**B**) from 30 replicates. Asterisks denote a significant difference with the control according to Dunnett’s test (*p* < 0.05).

To see if AE had some positive effect on the regeneration of *N. benthamiana* leaf discs, various concentrations of AE (5, 10, 50, or 100 mg L^−1^) were added to the MS regeneration medium (Figure 1). Although no significant differences were observed, the percentage of regeneration averaged 80% from the control (MS medium) while in the combination, MS with AE almost reached 100% regardless of the concentration used (Figure 1A). The number of regenerated buds per explant was enhanced by 3.5 times when 5 and 10 mg L^−1^ were added to the MS regeneration medium and by 6.5 times and 4.5 times with 50 and 100 mg L^−1^, respectively (Figure 1B). An illustration of the positive effect of AE on *N. benthamiana* regeneration is shown in Figure 1C.

### 2.4. Effect of Ascophyllum nodosum Extracts on Regeneration of Prunus domestica

**Figure 2:**
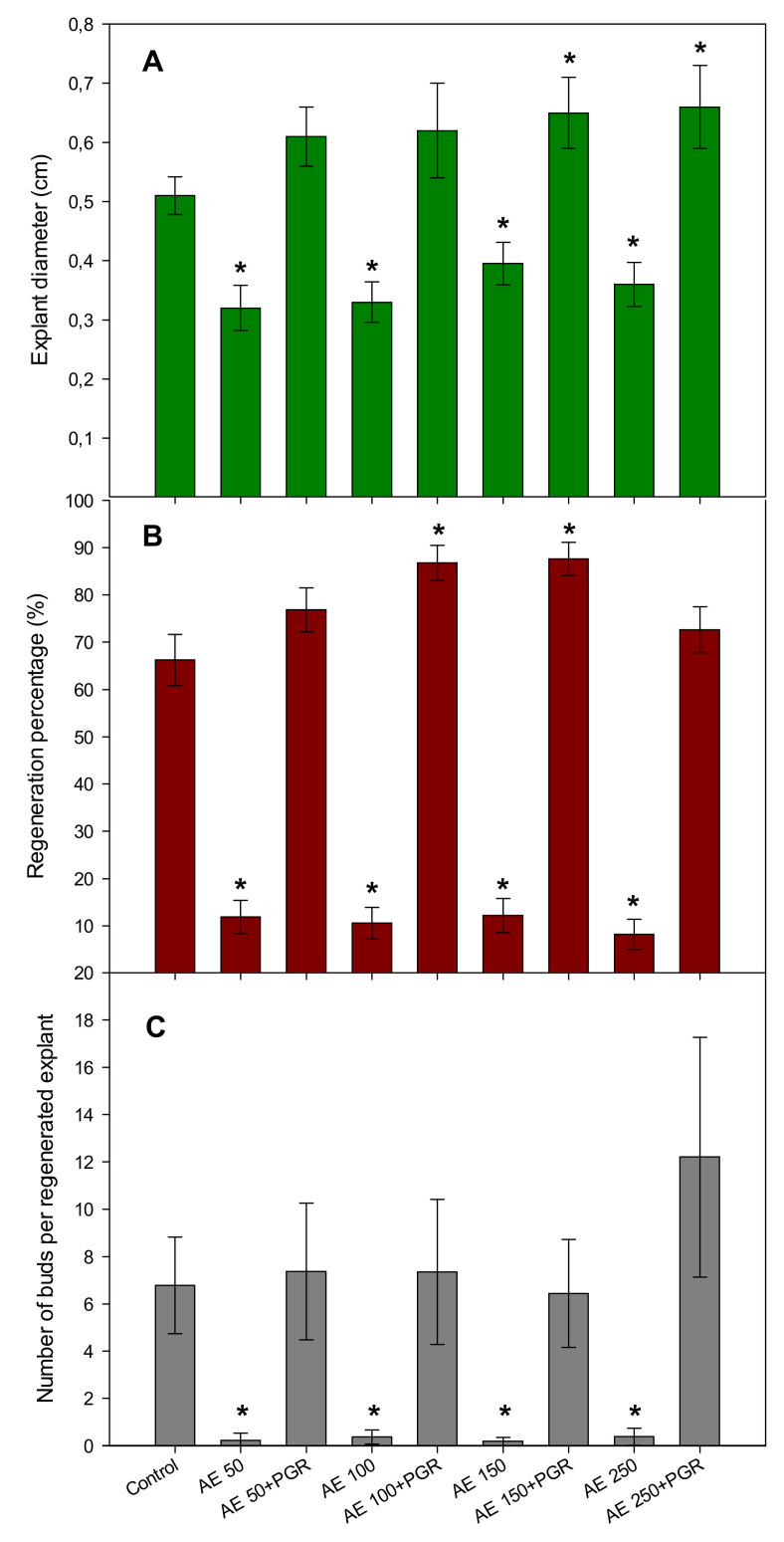
Effects of *Ascophyllum nodosum* extract (AE) at 50, 100, 150, and 200 mg L^−1^ alone or combined with plant growth regulators (PGR) on (**A**) the explant diameter, (**B**) the percentage of bud regeneration, and (**C**) the number of buds per explant regenerated from hypocotyl slices of *Prunus domestica* cv. Stanley. Data are percentages ± standard errors in (**A**) and means ± standard errors in (**B**) and (**C**) from 60 replicates. Asterisks denote significant difference with the control according to specific contrasts for regeneration percentages and according to the Dunnett’s test (*p* < 0.05) for explant diameter and the number of buds per explant.

To see if AE could affect organogenesis in woody plants, we used a ready laboratory protocol of regeneration from hypocotyl slices of plum. Hypocotyl segments (0.5 mm diameter) were allowed to regenerate in the control medium consisting of SRM with TDZ and IBA as PGR and compared to those cultivated in the same medium in which PGR was replaced by 50, 100, 150, or 250 mg L^−1^ of AE. In another trial, the same concentrations of AE were added to the SRM medium containing PGR. The effect of AE was determined after four weeks of sub-culture by assessing the diameter of slices, the percentage of bud regeneration as well as the number of regenerated buds (Figure 2).

The increase of explant diameter is related to the quality of explants that are able to regenerate new buds. In the control medium where 100% synthetic PGR was added, the diameter of slices increased by 10 times, reaching 5 mm while in the medium in which PGR was substituted by AE, increase in diameter ranged only from five to six times. However, complementation of the control medium with AE increased the diameter of the slices by 12 to 13 times. Increases were significant when 150 or 250 mg L^−1^ of AE was added (Figure 2A). 

Adventitious shoot regeneration was observed by the naked eye from plum hypocotyl slices as the regeneration of new plum buds. The percentage of bud regeneration reached 66% in the control medium and a significant increase was observed when 100 and 150 were added to the medium, reaching the maximum value of 88%. When only AE were added to the medium, the percentage of regeneration fell to around 10% (Figure 2B). Similar results were found when the number of regenerated buds per disc was recorded (Figure 2C).

**Figure 3:**
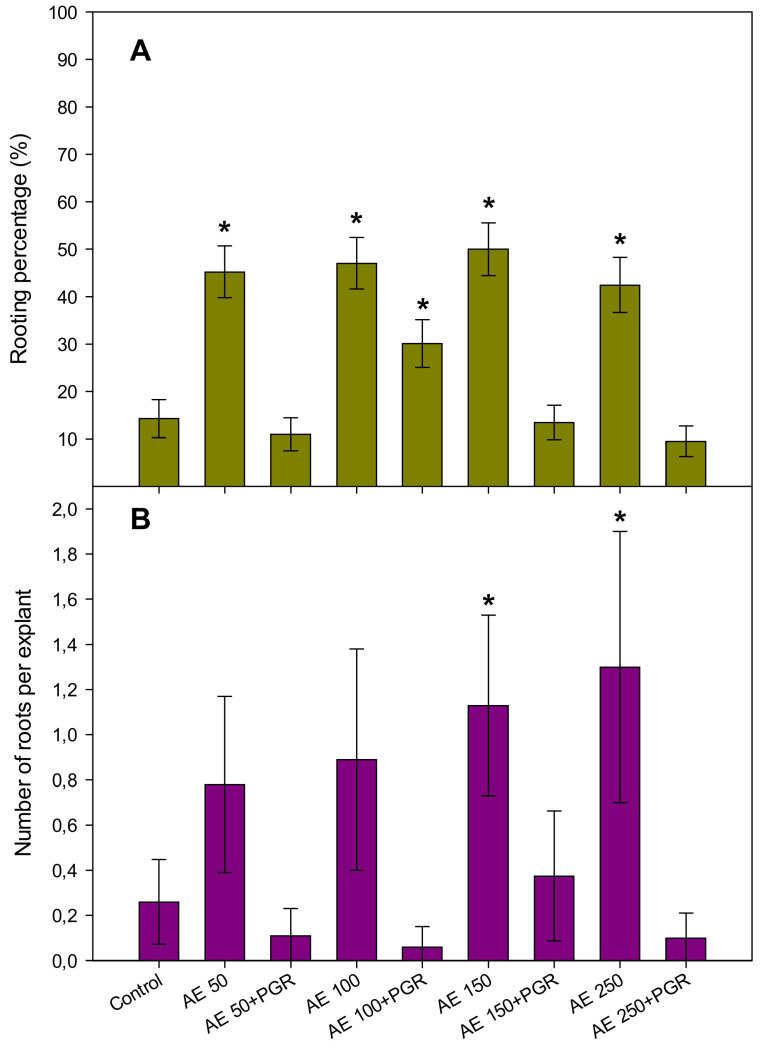
Effects of *Ascophyllum nodosum* extract (AE) at 50, 100, 150, 200 mg L^−1^ alone or combined with Plant Growth regulators (PGR) on (**A**) the percentage of rooting and (**B**) the number of roots per rooted explant from hypocotyl slices of *Prunus domestica* cv. Stanley. Data are percentages ± standard errors in (**A**) and means ± standard errors in (**B**) from 60 replicates. Asterisks denote significant difference with the control according to specific contrasts for rooting percentages and according to the Dunnett’s test (*p* < 0.05) for the number of roots per explant.

The effect of AE on rhizogenesis was also determined after four weeks of culture by assessing the percentage of rooting and the number of roots per slice (Figure 3). 

The percentage of rooted slices averaged 15% in the control medium and an increase was recorded when 100 mg L^−1^ of AE were added to the medium. The rooting percentage did not differ significantly from those slices regenerated when the medium was supplemented with the other AE concentrations. However, rooting was drastically increased when PGR were substituted by AE, reaching around 50% with 150 mg L^−1^ of AE (Figure 3A). The highest number of roots per explant was observed in the medium with 150 and 250 mg L^−1^ of AE alone (Figure 3B).

**Figure 4:**
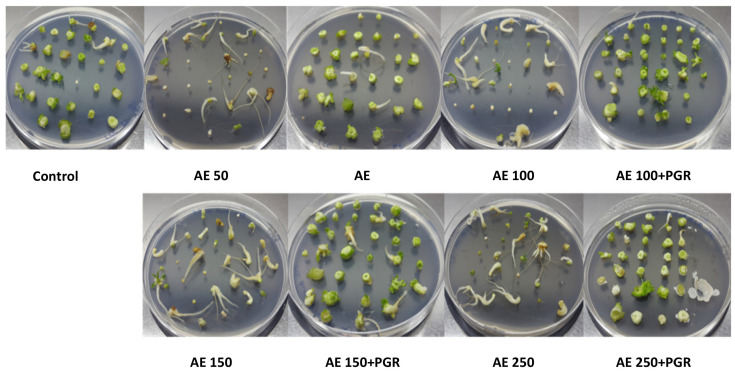
Photos showing regeneration of buds and roots from slices of hypocotyls of *Prunus domestica* cv. Stanley in medium consisting of *Ascophyllum nodosum* extract (AE) at 50, 100, 150 and 200 mg L^−1^ alone or combined with plant growth regulators (PGR).

A representative illustration of the effect of AE on shoot regeneration and rooting is shown in Figure 4, where the appearance of new buds and roots can be observed.

## 3. Discussion

This study was designed to verify if a commercial extract derived from *A. nodosum* could be valorized in the micropropagation and in vitro regeneration of higher plants using two model plants used routinely in our laboratory: the herbaceous plant *N. benthamiana* and the woody plant *P. domestica*. 

Our results showed that AE alone could not promote micropropagation of *N. benthamiana*, while it was capable to improve some growth parameters when combined with a half-strength MS medium. These results suggest that although AE was largely described as a source of naturally occurring nutrients that enhance crop health, nutrition, and quality, it did not contain sufficient macro- and micro-elements and vitamins to successfully complete the in vitro growth of *N. benthamiana*. However, we found that the addition of 100 mg L^−1^ AE to MS/2 enhanced root length. The positive effect of AE on rooting was reported in *A. thaliana* grown in vitro [36]. The effect of AE was even more evident in hypocotyl slices of *P. domestica* since substitution of PGR with AE enhanced rhizogenesis by at least three times. However, there is no additive effect between the AE and PGR. Treatments with AE enhanced root growth in lettuce and watermelon growing under greenhouse conditions as well as in lettuce and strawberry in field trials [37]. The rooting process of plants is a critical step for the establishment of new woody plants. The development of a large, robust root system is also essential for in vitro plant survival during the acclimation step. In this sense, the ability of the AE to promote rhizogenesis is very interesting for the successful production of plum plants.

There is also a clear effect of AE on the regeneration of *N. benthamiana* since percentages reached 100% and the number of regenerated shoots was promoted by at least 3.5 times higher than the control, while this effect was not significant for the regeneration of buds from plum. It should be noticed, however, that substitution of the PGR in the control with AE was sufficient to induce some plum buds, although at low levels. The difference of regeneration from these two species may rely on their endogenous levels of PGR and/or their interaction with those present in AE. Surprisingly, analysis of PGR composition of AE revealed that their levels were very low and some of them remained undetected. These results suggest that the effect of adding AE is not related to the amount of hormones found. Goñi et al. [38], by quantifying phytohormones present in these extracts, concluded that the levels of PGR in AE were not sufficient to cause a significant effect in *A. thaliana* following seaweed extract application, but the observed effects may have resulted from the modulation of biosynthesis, quantity, and ratios of the endogenously produced cytokinins, auxins, and abscisic acid metabolites, rather than from the exogenous phytohormones present within the extracts themselves. Indeed, they showed that the application of AE increased cytokinin concentrations in *A. thaliana* tissues because of the inhibition of the expression of genes involved in cytokinin catabolism and was reported by Hurtado et al. [39]. Then, it is possible that the levels of cytokinin induced in *N. benthamiana* were higher than those induced in plum and are probably behind the differential regeneration efficacy observed in our study.

As a whole, these results underline the synergistic effect of the AE and PGR treatments. Combinations of seaweed extract and PGR with MS medium have been already reported to increase adventitious shoot regeneration in tomato [31]. In addition, the use of AE in combination with PGR boosted micropropagation of the cultivated algae *Kappaphycus alvarezii* [34,40,41,42,43]. In a recent study Rayorath et al. [35] studied the effect of two *Ascophyllum nodosum* extracts with and without combination with PGR on micropropagation of the tropical red alga *Gracilaria blodgetti*. The authors indicated that propagules of this alga treated with concentrations of AE as low as 0.1 mg L^−1^ could contribute to the successful production of vegetative propagules as seed stock. In most studies performed for micropropagation and regeneration of seaweed, the concentration of AE used was low, usually under 5 mg L^−1^ [40,44]. However, in our experimental conditions, the use of low concentrations did not positively affect propagation of the two species (data not shown) and at least 5 and 50 mg L^−1^ of AE were needed for *N. benthamiana* and *P. domestica*, respectively. Such a discrepancy is not surprising and simply indicate that the performance of AE on the regeneration differs according to the two phylogenetic groups, while it is known that regeneration is even genotype-dependent. Fan et al. [6] showed that adding at least 100 mg L^−1^ of AE to spinach shoots cultivated in vitro enhanced biomass as well as several physiological changes. The elevated concentration of AE needed for regeneration and micropropagation agree with our finding that AE did not contain sufficient PGR and the associated effects may also rely on other metabolic components. However, according to Hurtado et al. [40], our seaweed extracts are rich in amino acids that could serve as PGR precursors [45,46]. It should be noticed that the algal extracts used in this study are rich in glutamic acid and aspartic acid, counting for more than 0.9 and 0.6% of their composition, respectively. Glutamic acid plays a key role as a signaling molecule involved in plant growth under normal or stressful conditions [47]. Aspartic serves as a central building block for many constituents including nucleotides and plant growth regulator hormones [48]. Amino acids are known as biostimulants that improve plant growth and yield and serve as hormone precursors [2,49] and exogenously applied amino acids have also been reported to play a critical role in in vitro plant tissue culture [50,51,52].

Although more studies based on other plant species and varieties, and quantification of endogenous levels of PGR within these plants are needed, these results pave the way of the possible use of commercial *Ascophyllum nodosum* extracts for in vitro mass propagation of higher plants.

## 4. Materials and Methods

### 4.1. Seaweed Extracts and Plant Materials

Seaweed extract used in this work was a powder extract derived from the brownish algae *Ascophyllum nodosum*. It was a gift from Acadian Seaplants Limited, Nova Scotia, Canada. Its physical and chemical characteristics are described in [40].

Two plant species were used as the plant material in this study: *N. benthamiana* and seedlings from *P. domestica* cv. Stanley.

### 4.2. Analysis of Plant Growth Regulators Present in Seaweed Extract 

Plant growth regulators were analyzed mostly as described by Albacete et al. [53]. Cytokinins like trans-zeatin (TZ), zeatin riboside (ZR), and 2-iP, and other plant growth regulators like ABA, 1-aminocyclopropane-1-carboxylic acid (ACC), IAA, JA, SA, and gibberellic acid (GA1, GA3, and GA4) were determined in AE using a high-performance liquid chromatography/mass spectrometry (HPLC/MS) system consisting of an Agilent 1100 Series HPLC (Agilent Technologies, Santa Clara, CA, USA), equipped with a micro-well plate autosampler and a capillary pump, connected to an Agilent Ion Trap XCT Plus mass spectrometer (Agilent Technologies, Santa Clara, CA, USA) using an electrospray interface. Samples, in triplicate, were passed through a SepPak Plus C18 cartridge (SepPak Plus, Waters, Millford, MA, USA).

### 4.3. In Vitro Micropropagation Experiments

*N. benthamiana* shoots were micropropagated every four weeks in half-strength Murashige and Skoog (MS/2) as the control medium for micropropagation. This medium includes salts and vitamins. Shoots were also sub-cultured in various concentrations of AE (20, 40, 60, 80, or 100 mg L^−1^) or in MS/2 supplemented with 20, 40, 60, 80, or 100 mg. L^−1^ of AE. All of the micropropagation media were supplemented with 3% sucrose, solidified with 0.7% of agar and their pH were adjusted to 5.8 before being autoclaved at 121 °C for 20 min. The cultures were incubated in the growth chamber at 25 °C, under cool white fluorescent tubes (55 μmol m^−2^ s^−1^), with a 16 h photoperiod. Experiments were conducted in three replicates per treatment; each treatment consisted of 20 explants. The length of roots and shoots as well as the diameter of shoots and the leaf area were recorded after four weeks of sub-culture.

### 4.4. In Vitro Regeneration Experiments

Regeneration assays were performed with excised leaf discs from *N. benthamiana* with three replicates and 10 explants per replicate. As the control, leaf discs were incubated on Petri dishes containing MS medium supplemented with 3% sucrose, 4.4 μM BAP, and 0.5 μM naphthalene acetic acid (NAA). Discs were also incubated on Petri dishes containing either a mixture of MS medium or in MS medium in which NAA was replaced with 5, 10, 50, or 100 mg L^−1^ of AE. The percentage of regeneration as well as the number of regenerating buds was determined four weeks later.

Fruits of the plum (*Prunus domestica* L.) cv. Stanley were collected at ripening; their kernels were extracted and treated with a 1% sodium hypochlorite solution to eliminate any remaining flesh and stored at 4 °C until use. The kernels were split open and the seeds extracted before disinfection following the previously published procedures [54]. Briefly, kernels were immersed in a 1% sodium hypochlorite solution with 0.02% Tween-20 for 20 min and rinsed four times with sterile distilled water. Disinfected seeds were soaked in sterile water overnight at 4 °C and used as the source of hypocotyl slices for the regeneration experiments. The coats of the disinfected seeds were removed with a scalpel. The radicle and the epicotyl were discarded, and then the hypocotyl was sliced into three cross-sections (0.5 mm). Hypocotyl segments were allowed to regenerate in the following shoot regeneration media: (i) control SRM medium consisting of three quarter-strength Murashige and Skoog (MS) salts, full-strength MS vitamins, 2% (w/v) sucrose, 0.7% (w/v) purified agar, and the plant growth regulators (PGR), which consist of 7.5 μM thidiazuron (TDZ) and 0.25 μM 3-indolebutyric acid (IBA); (ii) SRM where all PGR were substituted with 10, 100, or 250 mg. L^−1^ of AE; and (iii) complete SRM supplemented with 10, 50, 100, 150, or 250 mg. L^−1^ of AE. The pH of the media was adjusted to pH 5.8. The effect of AE was determined after three weeks of culture under a 16-h photoperiod (20–25 μmol photons m^−2^ s^−1^) at 23 ± 1 °C by assessing the diameter of slices, the percentage of bud regeneration as well as the number of regenerated buds. The percentage of rooting and the number of emerged root from slices were also determined.

### 4.5. Statistical Analyses

The effects of *Ascophyllum nodosum* extracts on the length of roots and shoots, on the diameter of shoot, on the leaf area, and on the number of regenerated buds of *N. benthamiana* were tested using ANOVA. Additionally, the diameter of plum slices and the number of regenerated buds per slice were analyzed with ANOVA. All means were compared to the control treatment by a Dunnett’s test (*p* < 0.05). Regeneration and rooting percentages were analyzed by using maximum likelihood ANOVA from CATMOD module in SAS [55] and specific contrasts were designed when necessary.

## Figures and Tables

**Table 1:** Concentration of various growth regulators in the *Ascophyllum nudosum* extract.

ACC	TZ	ZR	2-iP	GA1	GA3	GA4	IAA	ABA	JA	SA
NF	NF	NF	0.47	NF	NF	0.11	NF	0.52	NF	10.20

Concentrations of 1-aminocyclopropane-1-carboxylic acid (ACC), trans-zeatin (TZ), zeatin riboside (ZR), 2-isopentenyl adenine (2-ip), gibberellic acid (GA1, GA3 and GA4), indole-3-acetic acid (IAA), abscisic acid (ABA), jasmonic acid (JA), and of salicylic acid (SA) are given in ng mL^−1^. NF: Not found.

**Table 2:** Effect of *Ascophyllum nodosum* extract (AE) on the in vitro micropropagation parameters of *Nicotiana benthamiana*. MS/2 = with a half-strength MS medium; 20, 40, 60, 80, 100 = concentrations of AE ranging from 20 to 100 mg L^−1^. Values are means ± standard errors.

Culture Media	Shoot Length (mm)	Shoot Diameter (mm)	Root Length (mm)	Leaf Area (mm^2^)
MS/2	25.0 ± 0.7	1.34 ± 0.06	5.3.0 ± 2.0	363 ± 30
MS/2 + 20 AE	31.1 ± 1.5 *	1.30 ± 0.11	54.0 ± 4.5	402 ± 39
MS/2 + 40 AE	34.7 ± 1.9 *	1.40 ± 0.12	61.3 ± 4.0	378 ± 32
MS/2 + 60 AE	30.8 ± 2.3 *	1.40 ± 0.12	5.67 ± 2.1	404 ± 43
MS/2 + 80 AE	31.7 ± 1.8 *	1.20 ± 0.07 *	5.92 ± 4.6	381 ± 36
MS/2 + 100 AE	24.4 ± 2.5	1.04 ± 0.04 *	67.1 ± 3.7 *	244 ± 53 *
20 AE	9.2 ± 1.5 *	1.00 ± 0.03 *	48.3 ± 6.0	60 ± 8 *
40 AE	9.2 ± 0.8 *	1.00 ± 0.04 *	38.3 ± 5.4 *	61 ± 7 *
60 AE	10.8 ± 0.8 *	0.96 ± 0.04 *	25.0 ± 4.3 *	48 ± 3 *
80 AE	10.0 ± 0.7 *	1.00 ± 0.04 *	38.3 ± 6.0 *	59 ± 10 *
100 AE	11.7 ± 1.1 *	0.92 ± 0.05 *	38.3 ± 6.5 *	63 ± 13 *

* Asterisks denote significant difference with the control (MS/2) according to a Dunnett’s test (*p* < 0.05) among treatments for each parameter.

## Data Availability

Data are contained within the article.
